# Lysophosphatidic acid modulates ovarian cancer multicellular aggregate assembly and metastatic dissemination

**DOI:** 10.1038/s41598-020-67565-7

**Published:** 2020-07-02

**Authors:** Yuliya Klymenko, Brandi Bos, Leigh Campbell, Elizabeth Loughran, Yueying Liu, Jing Yang, Oleg Kim, M. Sharon Stack

**Affiliations:** 1 Department of Obstetrics and Gynecology, Indiana University School of Medicine, Indianapolis, IN USA; 2 Harper Cancer Research Institute, University of Notre Dame, 1234 N Notre Dame Ave., A 200 Harper Hall, South Bend, IN 46617 USA; 3 Department of Chemistry and Biochemistry, University of Notre Dame, Notre Dame, IN USA; 4 Department of Cell and Developmental Biology, University of Pennsylvania Perelman School of Medicine, Philadelphia, PA USA

**Keywords:** Cancer microenvironment, Gynaecological cancer

## Abstract

Epithelial ovarian cancer (EOC) metastasis occurs by exfoliation of cells and multicellular aggregates (MCAs) from the tumor into the peritoneal cavity, adhesion to and retraction of peritoneal mesothelial cells and subsequent anchoring. Elevated levels of lysophosphatidic acid (LPA) have been linked to aberrant cell proliferation, oncogenesis, and metastasis. LPA disrupts junctional integrity and epithelial cohesion in vitro however, the fate of free-floating cells/MCAs and the response of host peritoneal tissues to LPA remain unclear. EOC MCAs displayed significant LPA-induced changes in surface ultrastructure with the loss of cell surface protrusions and poor aggregation, resulting in increased dissemination of small clusters compared to untreated control MCAs. LPA also diminished the adhesive capacity of EOC single cells and MCAs to murine peritoneal explants and impaired MCA survival and mesothelial clearance competence. Peritoneal tissues from healthy mice injected with LPA exhibited enhanced mesothelial surface microvilli. Ultrastructural alterations were associated with restricted peritoneal susceptibility to metastatic colonization by single cells as well as epithelial-type MCAs. The functional consequence is an LPA-induced dissemination of small mesenchymal-type clusters, promoting a miliary mode of peritoneal seeding that complicates surgical removal and is associated with worse prognosis.

## Introduction

With > 14,000 fatal cases in the United States and ~ 152,000 deaths worldwide per annum, ovarian cancer invariably remains the most lethal malignancy of the female reproductive system^1–3^. Predominantly (~ 90% of cases) affected by the most aggressive type of ovarian cancer—epithelial ovarian carcinoma (EOC), women usually present with widespread intraabdominal metastases at primary diagnosis, resulting in poor disease prognosis. Importantly, while surgical tumor debulking and standard platinum/taxane-based chemotherapy in such patients are initially efficacious, most women ultimately develop disease recurrence in the form of multi-drug resistant intraperitoneal metastasis, resulting in a 5-year relative survival rate of only 30%^4, 5^.

In a multi-step EOC intraperitoneal (transcoelomic) metastasis process, ovarian cancer cells escape the primary tumor individually or in cell:cell adherent cohorts by direct desquamation into the abdominal cavity. Buildup of malignant ascitic fluid further expedites intraperitoneal transport as anoikis-resistant single cells and multicellular aggregates (MCAs, or spheroids). The latter ultimately colonize and retract the mesothelial monolayer of peritoneally-lined abdominal organs, anchor in the submesothelial extracellular matrix (ECM) and proliferate to form metastatic nodules^6–8^. Formation of metastatic cells into MCAs is confirmed by their abundance in EOC patients’ effusions and in ascitic fluids collected from mice bearing intraperitoneal xenografts (our unpublished observations) and is considered one of the crucial steps ensuring successful metastatic dissemination^9–11^. Studies indicate that MCAs are more anoikis-, radio- and chemoresistant in comparison with single cells^8, 12–15^. MCAs also exhibit a higher propensity for immune evasion^16, 17^ and cancer stem cell potential^18–20^, as well as enhanced capacity for mesothelial clearance and ECM invasion at the secondary metastatic site^21, 22^. However, current understanding of the factors that control the cell–cell aggregation process into a multicellular metastatic unit and regulation of its subsequent fate remains largely elusive. Multiple molecules have been reported to impact ovarian cancer MCA generation and pro-metastatic behavior, including integrins^23^, cadherins^21, 24^, fibronectin^25^, matrix metalloproteinases^26^, CD44^27^, CDC25A^28^, angiotensin II^29^, nectin-4^30^ and others. Most importantly, modulation of the expression or activity of these proteins altered MCA clustering and impacted their pro-metastatic properties. Therefore, elucidating molecular mechanisms influencing MCA generation and dynamics may identify strategies to impair MCA formation and survival and thereby favorably reduce metastatic seeding.

During EOC transcoelomic dissemination, ovarian cancer cells and MCAs are subjected to a variety of external signals prevalent in the ascitic microenvironment. Lysophosphatidic acid (LPA) is a ubiquitous bioactive growth factor-like phospholipid that acts through a subfamily of G-protein coupled cell surface LPA-specific receptors (LPARs), evoking proliferative, adhesive/migrative/invasive and pro-survival responses in cells^31–34^. LPA is prevalent in the malignant ascites of EOC patients (1–80 μM) as compared to effusions from healthy individuals and women with benign ovarian neoplasms or non-malignant ascitic transudate^31, 35–37^. As opposed to normal ovarian epithelial cells which do not produce LPA at levels significant enough to stimulate aberrant receptor activation^38^, in EOC constitutive production of LPA by both ovarian cancer cells and mesothelial cells of the peritoneum^38, 39^ increases EOC cell adhesive, migratory and invasive properties, colony formation in vitro as well as tumorigenesis/metastasis in vivo^39–41^.

Multiple studies report a variety of mechanisms through which LPA triggers epithelial-to-mesenchymal transition (EMT)^7^. For example, it has been postulated that autotaxin, an enzyme responsible for production of LPA from lysophosphatidylcholine, can maintain an ovarian cancer stem cell population through an autocrine mechanism^42^. However, the role of LPA in modulating the intraperitoneal dynamics of MCAs remains understudied. Our group has previously reported time- and dose-dependent LPA-mediated disruption of EOC cell–cell connections through MMP-9-catalyzed E-cadherin extracellular domain cleavage resulting in loss of cell junctional integrity and epithelial cohesion, which could be restored by LPAR blocking^43^. Subsequently, we have demonstrated that LPA-treated cells and MCAs release a large cohort of proteins^7, 44^. In the current study we seek to comprehensively characterize the contribution of LPA to regulation of EOC MCA dynamics and peritoneal colonization through evaluating early adhesive events in peritoneal seeding.

## Results

### LPA decreases EOC cell aggregation capacity

**Figure 1:**
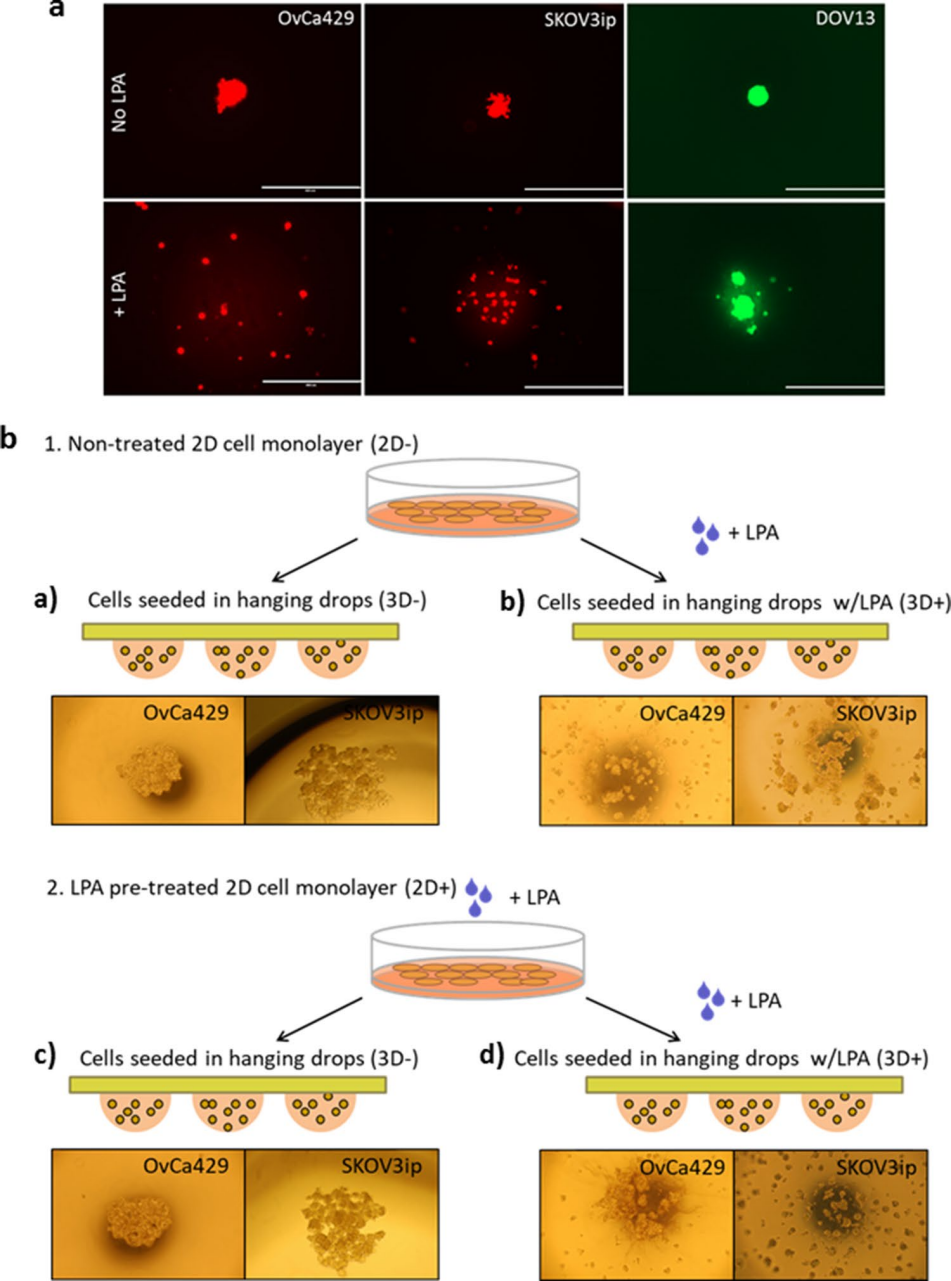
Exposure to LPA impairs ovarian cancer MCA assembly. (**A**) Fluorescently tagged (RFP or GFP) human EOC cells were seeded in 20 µl hanging drops at a 100,000 cell/ml concentration with or without 80 µM LPA (Cayman Chemical, Ann Arbor, MI) and incubated for 48–72 h. Imaging was then performed using AMG EVOS fluorescent microscope, scale bar = 400 µm. (**B**) Schematic and microscopic representation of MCAs generated under different LPA treatment conditions: EOC cells were grown in TC dishes until 60–70% confluence, incubated with or without 80 µM LPA for 24 h, harvested, seeded in 20 µl hanging drops at a 100,000 cell/ml concentration with or without 80 µM LPA. 4 different LPA treatment regimens were used: (a) cells were not treated with LPA in TC dishes and hanging drops (2D − 3D−); (b) cells were treated in hanging drops only (2D − 3D+); (c) cells were treated in TC dishes only (2D + 3D−); (d) cells were treated both in TC dishes and in hanging drops (2D + 3D+). After 48–72 h MCAs were imaged under light microscope (× 10).

We previously demonstrated that MCAs generated from epithelial- and mesenchymal-type ovarian cancer cells possess dramatically different phenotypes, ranging from loosely adhesive clusters (formed by purely epithelial-type E-cadherin expressing EOC cells) to highly cohesive solid spheroids (formed by purely mesenchymal-type N-cadherin-expressing EOC cells)^24^. To address the potential effect of LPA on MCA formation, in the current study we utilized an epithelial-type OvCa429 cell line, a mesenchymal-type DOV13 cell line and the highly mesenchymal SKOV3ip cell line that was selected for in vivo aggressive behavior. When incubated in hanging drops, OvCa429, DOV13 and SKOV3ip single cells readily assembled into a single MCA per drop. However, in the presence of LPA (80 μM), all three cell lines exhibited decreased aggregation capacity and formed multiple smaller clusters (Fig. 1A, Supplemental Fig. 1).

As our group has previously reported disruption of cell–cell junctions in cell monolayers by LPA, promoting their dissemination^43^, here we sought to further understand whether the observed alterations in MCA assembly rely on the continued presence of LPA in suspension. Four different LPA treatment regimens were implemented (Fig. 1B): (a) EOC cells grown as two-dimensional (2D) monolayers and subsequently seeded to grow three-dimensionally (3D) in hanging drops were left untreated (designated ‘2D − 3D−’); (b) EOC cells were grown in monolayers without LPA and further treated with 80 μM LPA in hanging drops (‘2D − 3D+’); (c) EOC cells were grown in monolayers with 80 μM LPA and subsequently incubated without LPA in hanging drops (‘2D + 3D−); (d) EOC cells were treated with 80 μM LPA both in monolayers and hanging drops (‘2D + 3D−’). As observed via light microscopy (Fig. 1B), OvCa429 and SKOV3ip EOC cells demonstrated impaired aggregation under 2D − 3D+ and 2D + 3D+ LPA treatments in comparison with the control 2D − 3D− condition. Interestingly, however, both OvCa429 and SKOV3ip cells incubated under 2D + 3D− LPA treatment exhibited avid MCA formation similarly to that of fully untreated 2D − 3D− control, suggesting that the effects of pre-treatment are reversible.

### LPA alters EOC cell/MCA surface ultrastructure

**Figure 2:**
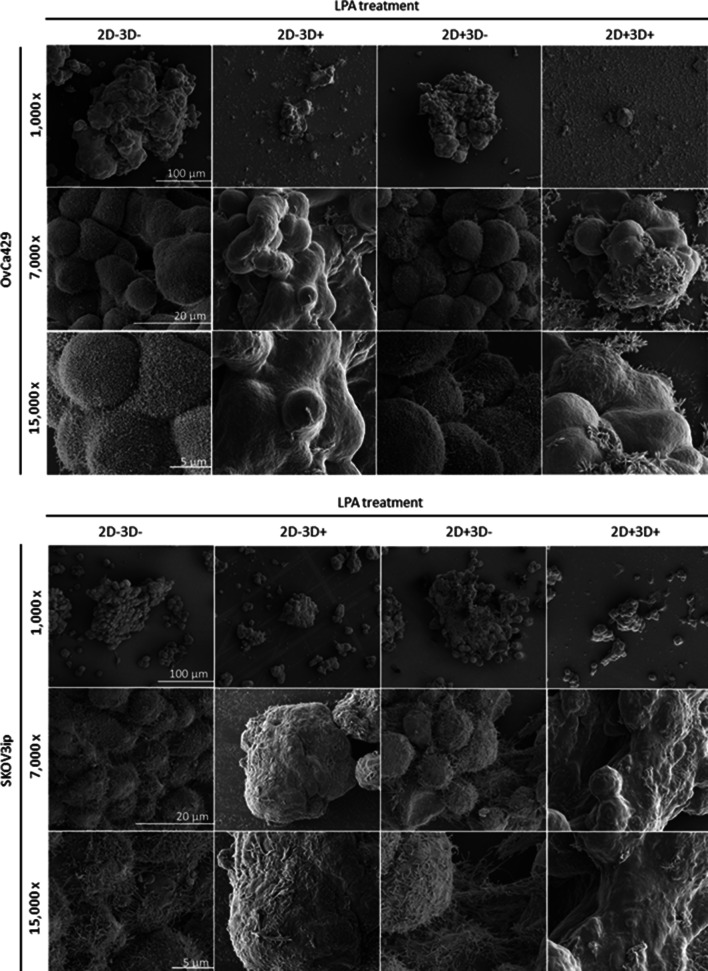
LPA reversibly alters EOC cell/MCA surface morphology. MCAs were generated from OvCa429 and SKOV3ip cells, as indicated, via the hanging drop method using LPA treatment conditions outlined in Fig. 1B; processed for SEM as detailed in “Methods”; and examined using FEI-Magellan 400 field emission SEM. Representative images were taken at × 1,000, × 7,000, and × 15,000 magnifications (scale bars as indicated), arrows depict sample inclusions released from cell surface.

To further decipher the effect of LPA on ovarian cancer MCA morphology, MCAs generated using the four LPA treatment regimens described above were visualized via SEM (Fig. 2). Remarkable differences in surface ultrastructure were observed correlating with LPA-treatment protocol. While untreated 2D − 3D− aggregates displayed high level of clustering, cell surface area and complexity (uniform microvilli coverage of OvCa429 MCAs and pronounced invasive filopodia coverage of metastatically aggressive SKOV3ip MCAs), poorly assembled 2D − 3D+ and 2D + 3D+ LPA-treated aggregates exhibited smooth cell surfaces with complete loss of protrusions in both cell types (Fig. 2). Inclusions in the samples were also observed, likely representing material released from cell surface in the presence of LPA (Fig. 2, arrows). To evaluate the nature of these inclusions, a comprehensive chemical analysis by multiplex coherent anti-Stokes Raman scattering imaging combined with mass spectrometry was performed. The results confirmed that the proteinaceous nature and composition of these structures were matched to the surface of the LPA-exposed cells/MCAs^44^. In support of the reversible nature of the ultrastructural changes, scanning electron micrographs of 2D + 3D− LPA-treated OvCa429 and SKOV3ip MCAs depicted surface morphology patterns identical to those of corresponding fully untreated MCAs (Fig. 2).

### LPA reduces EOC cell adhesion to peritoneal tissues

**Figure 3:**
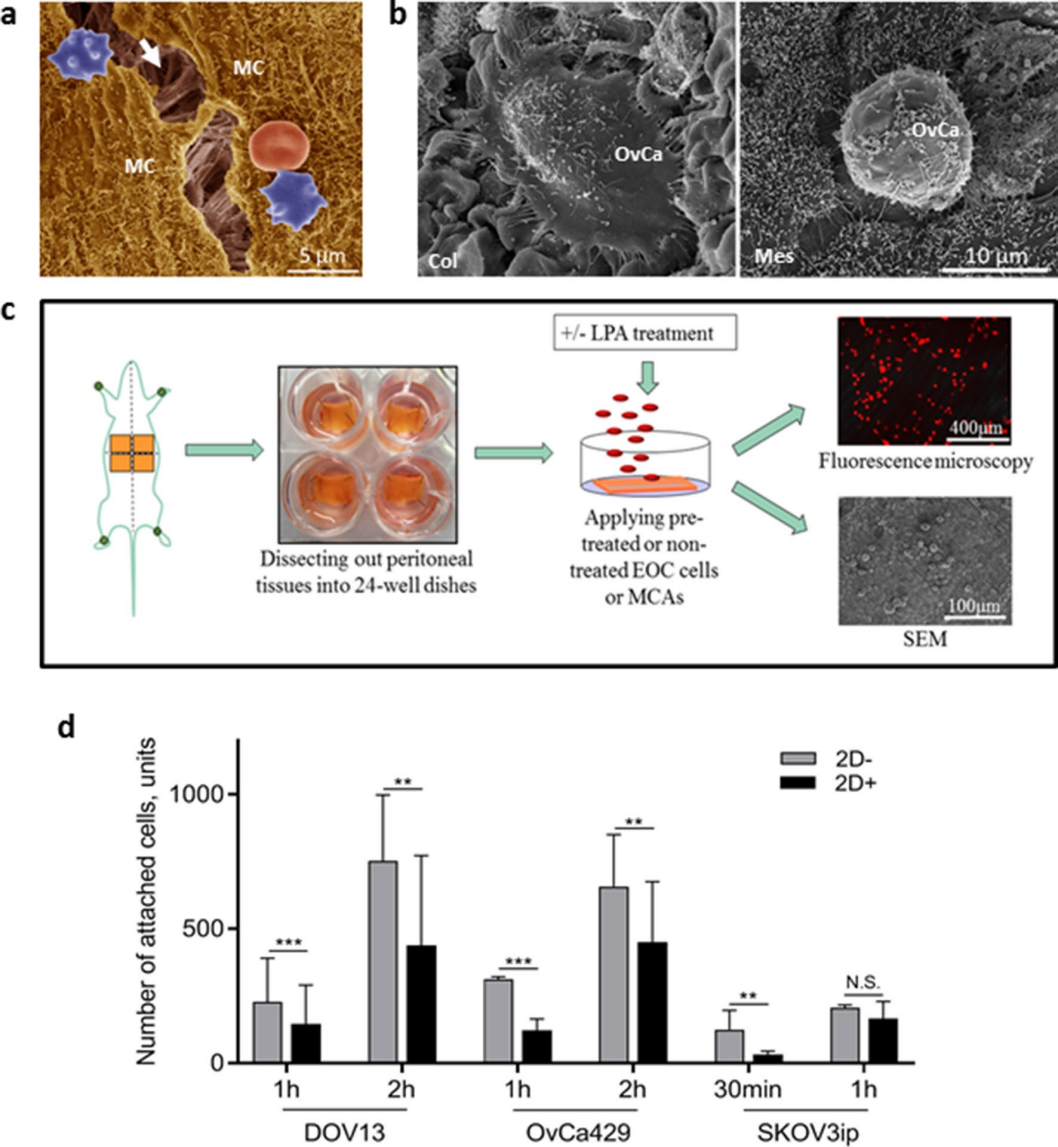
LPA attenuates EOC cell adhesion to murine peritoneal tissues. (**A**) Pseudo-colorized SEM micrograph of a C57BI/6 mouse peritoneal tissue (dissected, processed for SEM and imaged as detailed in “Methods”) visualizes microvilli-covered mesothelial cells (MC, yellow) which form a monolayer atop underlying 3D collagen-rich submesothelial extracellular matrix (arrow). The integrity of the MC monolayer in the current micrograph was surgically compromised during the dissection procedure and allows visualization of the underlying ECM collagen fibers (arrow). Red blood cells—erythrocytes (red) and echinocytes (purple)—are also in the field of view. (**B**) Scanning electron micrographs of ovarian cancer SKOV3ip cells seeded atop C57BI/6 mouse peritoneal explants (dissected, cell-seeded, SEM-processed and imaged as detailed in “Methods”) display adhesion and dispersal of an individual ovarian cancer (OvCa) cell on top of naked collagen (Col) vs. mesothelium (Mes) after 1 h of seeding. (**C**) Schematic representation of the peritoneal adhesion assay: fluorescently labeled OvCa429, DOV13, and SKOV3ip cells, pre-treated or not treated with 80 µM LPA (2D+ or 2D−), were seeded on dissected and pre-pinned C57Bl/6 murine peritoneal explants and incubated for times indicated; tissues were then examined using EVOS fluorescence microscope or SEM FEI-Magellan 400 as detailed in “Methods”; (**D**) quantitative analysis of images was performed in ImageJ and statistical significance (defined as **p < 0.01, ***p < 0.001) was calculated using a two-sided Mann–Whitney U test. The data are presented as mean ± SD. Scale bars: as indicated.

As an early event in metastatic dissemination, EOC cells first encounter a monolayer of mesothelial cells that overlie a thin basement membrane and serve as an active barrier between tumor cells and the underlying submesothelial collagen-rich 3D ECM (Fig. 3A). The integrity of this mesothelial cell monolayer is key to regulation of metastatic dissemination and research indicates that cancer cells adhere more readily to areas of the peritoneum exhibiting cleared mesothelium^45, 46^. By examining ex vivo peritoneal tissue explants under SEM, we commonly observed that individual SKOV3ip cells rapidly adhered to and spread upon peritoneal zones with exposed collagen (Fig. 3B, left), in contrast to cancer cells attaching directly to mesothelium, which did not exhibit spreading (Fig. 3B, right). To delineate the potential impact of LPA on the ability of EOC cells and MCAs to colonize mesothelium, adhesion to intact peritoneal explants was quantitatively evaluated^24^ using fluorescently labeled cells or MCAs (Fig. 3C). LPA pre-treatment of EOC cell 2D monolayers significantly decreased adhesion of individual cells to peritoneal explants (Fig. 3D–F). It is interesting to note that differences between treated and untreated highly aggressive SKOV3ip cells were observed at early time point, but by 1 h, all of the cells were fully adherent.

### LPA attenuates EOC MCA-to-mesothelium adhesion and mesothelial clearance

**Figure 4:**
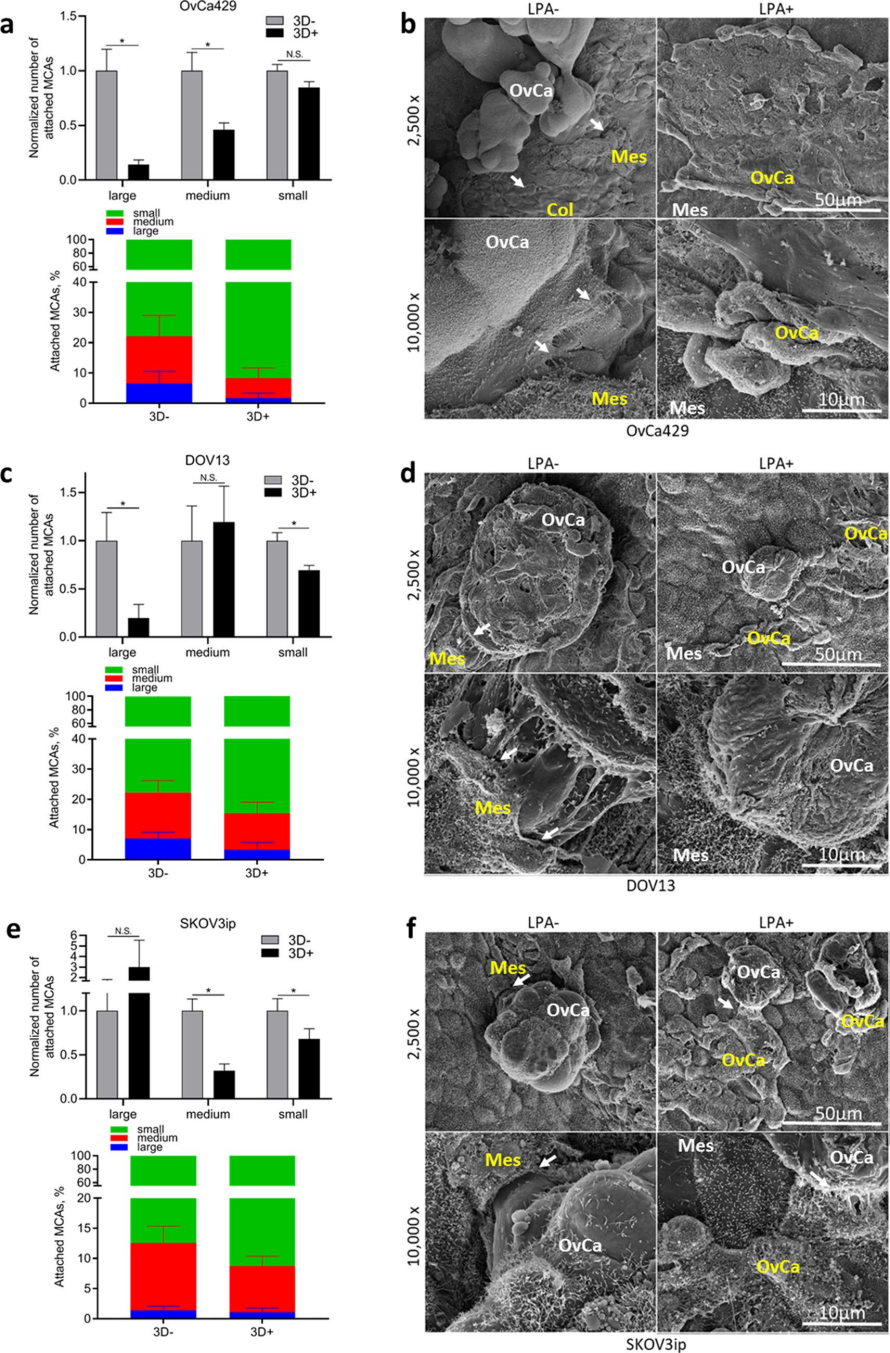
LPA regulates MCA peritoneal adhesion and mesothelial clearance activities. MCAs were generated from fluorescently labeled OvCa429, DOV13, and SKOV3ip cells via the hanging drop method with or without 80 µM LPA (3D+ or 3D−, respectively), seeded atop C57Bl/6 murine peritoneal explants and incubated, as indicated in “Methods”; tissues were then examined using EVOS fluorescence microscope or SEM FEI-Magellan 400, as stated in “Methods”; (**A**,**C**,**E**) quantitative analysis of fluorescent images was performed in ImageJ and statistical significance (defined as *p < 0.05; *N.S.* non-significant) was calculated using a two-sided Mann–Whitney U test. The data are presented as mean ± SEM (n > 35); (**B**, **D**, **F**) representative scanning electron micrographs of MCAs attached to peritoneal explants were taken at × 2,500 and × 10,000 magnifications (scale bars as indicated). *Col (red)* compromised/naked collagen, *Mes (white)* intact mesothelium, *Mes (red)* compromised/cleared mesothelium, *OvCa (white)* competent ovarian cancer MCA, *OvCa (red)* compromised/dead ovarian cancer MCA, white arrows indicate places of active mesothelial clearance by EOC MCA leading cells.

As reported above (Figs. 1, 2), LPA impedes aggregation of cells into larger MCAs. To access the pro-metastatic behaviors of EOC MCAs at the peritoneal site via the peritoneal adhesion assay (described in Fig. 3B), EOC cells were incubated in hanging drops with or without 80 μM LPA (3D+ or 3D−, respectively) for 48 h and then an equal number of drops were collected for each condition and reseeded onto live murine peritoneal explants. Evaluation of attached MCAs using fluorescence microscopy revealed altered adhesion patterns in all cell lines (Fig. 4A,C,E, greyscale panels). Overall analysis of the size distribution of adhered MCAs or spheroids demonstrated an LPA-induced shift towards a miliary pattern of peritoneal seeding (Fig. 4A,C,E, colored panels).

Upon examination of peritoneal explants under SEM, noticeable differences in MCA dispersal and mesothelial clearance function were observed. In particular, untreated OvCa429, DOV13 and SKOV3ip MCAs displayed lateral dispersal of MCA leading cells, migration underneath mesothelium, disruption and clearance of damaged mesothelial layer and exposure of underlying collagen (Fig. 4B,D,F, left panels) within 2 h of seeding. In striking contrast, LPA-treated epithelial-type OvCa429 MCAs fully segregated on top of uncompromised mesothelial monolayer and underwent full aggregate destruction, as evidenced by disintegration of ovarian cancer cell membrane and loss of intracellular cytoplasmic content (Fig. 4B, right panel). In turn, LPA-treated mesenchymal-type DOV13 and far-mesenchymal-type SKOV3ip MCAs exhibited partial segregation and cell death atop mesothelial layer similar to that of OvCa429; nevertheless, some of DOV13 and SKOV3ip cells survived and partially retracted the mesothelium, although to a lesser level relative to untreated MCAs (Fig. 4D,F, right panels).

### LPA modulates host peritoneal tissue ultrastructure

**Figure 5:**
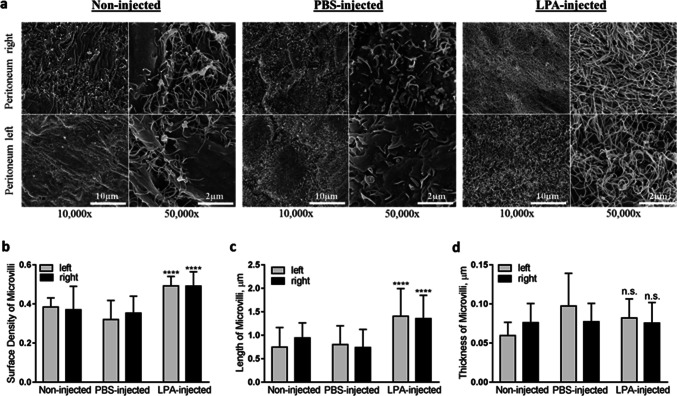
LPA modulates host peritoneal tissue ultrastructure. (**A**) C57Bl/6 mice were intraperitoneally administered 1 × PBS (1 ml), 80 μM LPA (1 ml) or left non-injected for 5 consecutive days; murine peritoneal tissues were then dissected, processed for SEM as detailed in “Methods”; and examined using FEI-Magellan 400 field emission SEM. Representative images were taken at × 10,000, and × 50,000 magnifications (scale bars as indicated). Quantitative analysis of mesothelial cell surface microvilli (**B**) density, (**C**) length, and (**D**) thickness was assessed using standard Fiji open source software. The data are presented as mean ± SD, n = 250. Statistical significance (defined as ****p < 0.0001; n.s. non-significant) was calculated using a two-sided Mann–Whitney U test.

While a multitude of studies focus on the role of LPA in promoting cancer cell oncogenic functions, the impact on host peritoneal tissues remains uninvestigated. To address this question, C57BI/6 female mice were injected intraperitoneally daily with LPA or PBS for 5 consecutive days or left un-injected. Subsequent SEM examination of peritoneal explants revealed that non-injected and PBS-injected mice shared similar peritoneal morphology. In contrast, LPA-injected mice demonstrated significantly enhanced mesothelial surface area and complexity as evidenced by augmented density and length (but not thickness) of mesothelial cell surface microvilli (Fig. 5, Supplemental Fig. 2). Of note, one time short-term (30 min) intraperitoneal administration of LPA (80 μM) did not cause ultrastructural changes in murine peritoneal tissues compared to PBS-injected or non-injected controls (data not shown), suggesting that this is not an acute response.

### LPA restricts mesothelial susceptibility to EOC single cells and epithelial-type EOC MCAs

**Figure 6:**
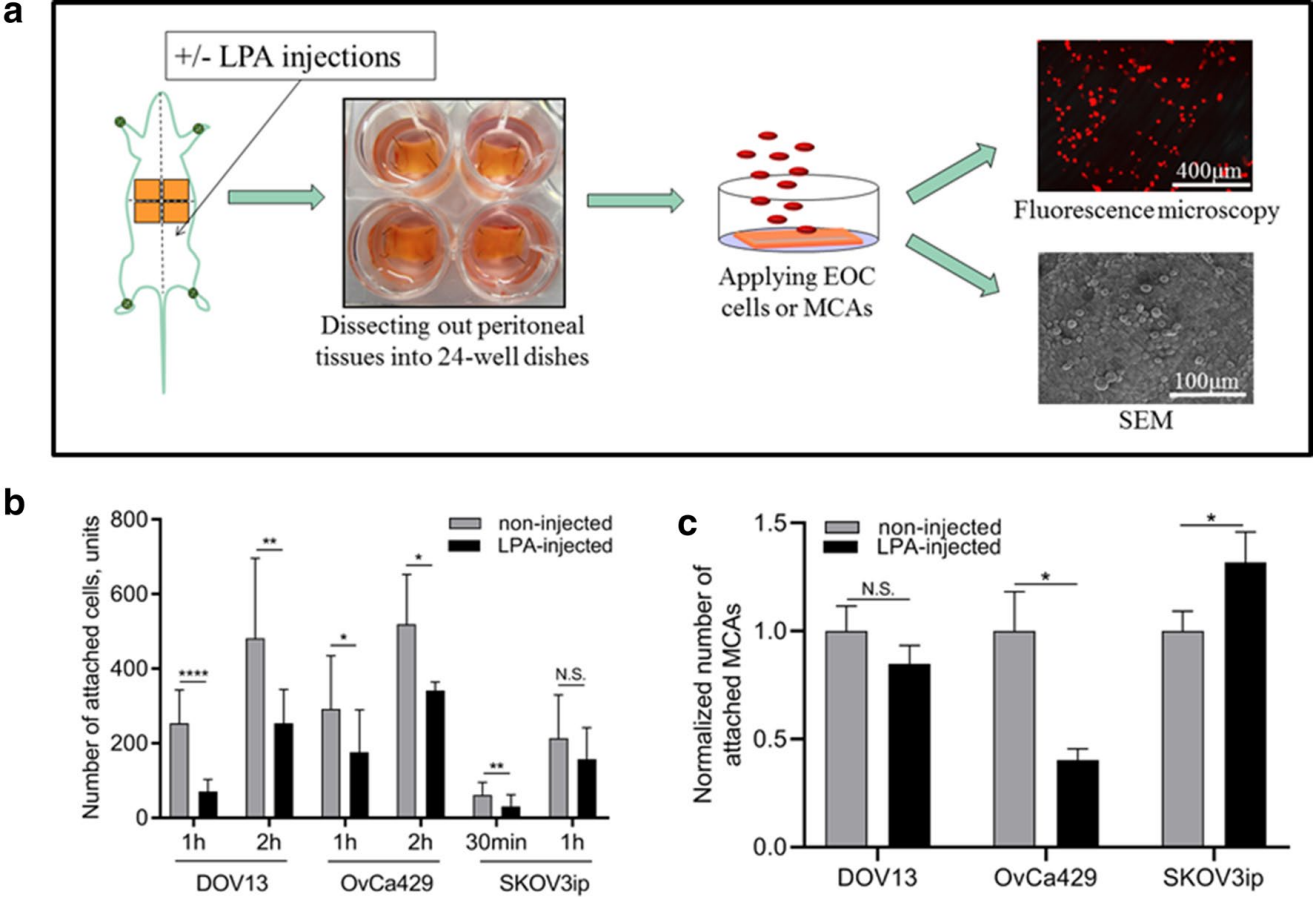
LPA alters mesothelial susceptibility to colonizing EOC cells and MCAs. (**A**) Schematic representation of the peritoneal adhesion assay workflow: C57Bl/6 mice were intraperitoneally administered 80 μM LPA (1 ml) or left non-injected for 5 consecutive days; murine peritoneal tissues were then dissected, fluorescently labeled OvCa429, DOV13, or SKOV3ip cells or pre-generated MCAs (via hanging drop method), were seeded atop and incubated as detailed in “Methods”; tissues were then examined using EVOS fluorescence microscope or SEM FEI-Magellan 400; (**B**,**C**) quantitative analysis of images was performed in ImageJ and statistical significance (defined as *p < 0.05, **p < 0.01, ****p < 0.0001, *N.S.* non-significant) was calculated using a two-sided Mann–Whitney U test. The data are presented as mean ± SD (**B**) and mean ± SEM, n > 35 (**C**).

To further evaluate functional consequences of LPA-induced ultrastructural changes in tumor cell and mesothelial tissue ultrastructure, an ex vivo peritoneal adhesion assay was employed, wherein fluorescently labeled EOC single cells or MCAs were applied to peritoneal explants obtained from un-treated or LPA-treated (5 days) mice and evaluated by fluorescence or scanning electron microscopy (Fig. 6A). Adhesion of single cells to peritoneal tissues from LPA pre-treated mice was significantly reduced regardless of the epithelial or mesenchymal phenotype of the cell (Fig. 6B). A similar reduction in adhesion of MCAs formed from epithelial phenotype OvCa429 cells to LPA pre-treated peritoneum was observed (Fig. 6C). Adhesion of MCAs formed from mesenchymal-type cells was either enhanced (SKOV3ip) or unaffected (DOV13) (Fig. 6C).

## Discussion

Despite considerable advances in therapeutic management, EOC has persisted as the deadliest gynecological malignancy for decades, thereby clearly illustrating the unmet need for a more fundamental understanding of the basic biology that underlies disease progression, aggressive metastatic dissemination and recurrence. LPA is abundant in 90% of women with stage I ovarian cancer and 100% of patients with late stages of the disease^47^. Hence, ovarian cancer cells and MCAs are subjected to LPA signaling at all steps of EOC transcoelomic dissemination. Multiple studies clearly implicate LPA in ovarian tumorigenesis, EMT regulation, enhanced cancer cell adhesion, migration/invasion, proliferation, and regulation of ovarian cancer stem cell potential^7, 39–42, 48^. The current study reports the unexpected finding that LPA negatively impacts the assembly and implantation of MCAs.

MCAs are now commonly recognized as EOC metastatic units that possess enhanced survival relative to single cells due to augmented anoikis- and therapeutic resistance and the capacity to sustain immune surveillance^8,12–14,16,18^. It was recently demonstrated^28^ that in comparison with cell monolayers, ovarian multicellular spheroids exhibit chemoresistance through aberrant activation of cell cycle-related pathways, halting cell cycle progression and suppressing platinum- or taxane-induced cell death. Moreover, clustering of detached cancer cells creates a hypoxic environment and triggers a hypoxia-inducible factor 1-alpha (Hif1α)-mediated metabolic switch that restricts reactive oxygen species accumulation. Conversely, disruption of cellular aggregates led to reactive oxygen-mediated apoptosis and reduced metastasis^22^.

Herein, we demonstrate that LPA promotes loss of EOC cell surface protrusions, impairs the ability to assemble into large MCAs/spheroids, and mitigates adhesion to peritoneal tissues. The functional consequence is an LPA-induced dissemination of small clusters, potentially promoting a miliary mode of peritoneal seeding that complicates surgical removal and is associated with worse prognosis. Experiments in this study have used concentrations of LPA demonstrated to be present in cancerous ascites fluid^31, 35–37^. We have previously shown that LPA disrupts E-cadherin-mediated junctional integrity in monolayer culture and this effect is blocked by the small molecule inhibitor Ki16425^48^. Moreover this was accompanied by gain of a mesenchymal phenotype in LPA-treated MCAs, characterized by loss of E-cadherin, gain of vimentin expression, rearrangement of F-actin stress fibers and punctate vinculin staining^48^. Clustering of β1 integrins was also observed, accompanied by an increase in the population of conformationally active β1 integrins, potentially activating signaling pathways that promote a more mesenchymal phenotype^7, 48^.

Cytoreductive surgery in patients with miliary intraperitoneal dissemination of advanced high grade serous ovarian carcinoma (HGSOC) is associated with a greater level of surgical complexity together with a higher residual disease score^49^. Furthermore, compromised MCAs with repressed peritoneal adhesion function may harness alternative metastasis patterns. In support of that, a recent study^27^ demonstrated that interference with ovarian cancer spheroid formation and inhibition of spheroid-to-mesothelial adhesion through targeting CD44 blocked mesenteric colonization but instead provoked unrestrained distant in vivo metastases in the thoracic cavity and liver.

An unanticipated finding of the current work is that LPA most effectively affects survival, mesothelial clearance and peritoneal implantation of epithelial-type OvCa429 MCAs^24^. A recent study^50^ outlined the necessity of E-cadherin as a survival factor for detached cancer cells and a requirement for successful distant metastatic re-colonization in multiple mouse and human models of breast carcinomas. Conversely, loss of E-cadherin triggered reactive oxygen-induced circulating tumor cell apoptosis and restricted early phases of metastatic seeding^50^. Our observation that LPA-treated OvCa429 MCAs exhibit dissociation and cell death on intact mesothelial tissues is consistent with these data and suggest a link to an LPA-induced loss of E-cadherin from OvCa429 cell/MCA surface^43^. Importantly, E-cadherin-negative DOV13^24^ and SKOV3ip^7^ MCAs displayed incomplete segregation and apoptosis while retaining partial capacity to further penetrate mesothelium, suggesting that mesenchymal-type E-cadherin-deficient cells/MCAs possess alternative survival mechanisms. In agreement with that is our mass spectrometry analysis^44^ that reports divergent lists of peptides released from OvCa429 and SKOV3ip MCAs by LPA, suggesting differential responses of epithelial- and mesenchymal-type cells to LPA-induced stress.

In addition to exploring the impact of LPA on EOC cell/MCA structure and function, the current study also addressed the response of host peritoneal tissues to this bioactive phospholipid in vivo and ex vivo. Our data indicated activation of mesothelial cells, as evidenced through alterations in their surface morphology with increased number and elongation of surface microvilli, which functionally resulted in mitigation of EOC cell and epithelial-type MCA adhesion. These results support a protective role of the mesothelium as a bioactive mechanical barrier against tumor cell peritoneal anchorage and colonization^21, 45, 46^. Notably, activated mesothelial cells successfully blocked adhesion of all three types of EOC single cells which is consistent with our previous observations of single cell-mesothelium interaction^21^. Meanwhile, LPA-mediated activation of mesothelial cells was efficient in suppressing epithelial-type OvCa429 MCA implantation, but was not sufficient to inhibit seeding of mesenchymal-type DOV13 and SKOV3ip MCAs. These results align with research indicating that ovarian cancer spheroids harnessing a mesenchymal gene signature possess an augmented capacity for mesothelial clearance^21, 51^. Together these data support a role for LPA in promoting the differential implantation of mesenchymal-type metastatic cell clusters at secondary metastatic sites and indicate that LPA-induced changes to both the tumor and the host are important in regulating adhesive events in metastasis. This is supported by clinical data showing that HGSOC patients with miliary intraperitoneal disease predominantly present lesions of mesenchymal molecular subtype^49^ and highlight a need for continued evaluation of anti-metastatic therapies targeting mesenchymal-type MCAs.

## Materials and methods

### Cell lines

DOV13 and OvCa429 are human ovarian cancer cell lines that have been obtained from Dr. RC Bast, MD Anderson Cancer Center, Houston TX. Cells were maintained in Minimal Essential Medium (MEM; Gibco, Big Cabin, OK) containing 10% Fetal Bovine Serum (FBS; Gibco), 1% Non-Essential Amino Acids (Corning Cellgro, Manassas, VA), 1% Penicillin/Streptomycin (Lonza, Allendale, NJ), 1% Sodium Pyruvate (Corning Cellgro), 0.1% Amphotericin B (Cellgro), and supplemented with 10 μg/ml Insulin (Gibco) for DOV13 cell medium only^21, 24, 26, 43^. Human EOC SKOV3ip cells were obtained from Dr. Katherine Hale (University of Texas M.D. Anderson Cancer Center, Houston, TX) and maintained in RPMI-1640 medium (Corning Cellgro), supplemented with 10% FBS, 1% l-glutamine (Gibco by Life Technologies), 1% sodium pyruvate, 1% Pen/Strep, 1% NEAA, 1% 4-(2-hydroxyethyl)-1-piperazineethanesulfonic acid (Gibco by Life Technologies), and 0.1% Amphotericin B^44^. Cell lines were tested and authenticated by Genetica DNA Laboratories using short tandem repeat (STR) DNA profiling and were found to be > 95% concordant^21, 24, 26, 43^.

RFP lentiviral vector (GenTarget, San Diego, CA) was used to generate fluorescently tagged OvCa429-RFP and SKOV3ip-RFP cell lines. GFP lentiviral vector (AddGene, Cambridge, MA) was utilized to create DOV13-GFP stable cell line. Lentiviral transductions were performed according to manufacturers’ protocols and successfully tagged cells were further selected via BD FACSAria III cell sorter^21^.

### MCA formation

Two-dimensional (2D) EOC cell monolayers were grown in tissue culture (TC) dishes and a hanging drop method was employed to form free-floating three-dimensional (3D) ovarian cancer MCAs^52,53^. LPA was purchased from Avanti Polar Lipids, Inc. (Alabaster, Alabama) and four different LPA treatment regimens were applied during MCA generation (Fig. 1): (1) cells remained untreated in both 2D TC dishes and 3D hanging drops (designated ‘2D − 3D−’); (2) cells were treated with LPA in hanging drops only (designated ‘2D − 3D+’); (3) cells were treated with LPA in TC dishes only (designated ‘2D + 3D−’); (4) cells were treated with LPA both in TC dishes and in hanging drops (designated ‘2D + 3D+’). Briefly, cells were grown in TC dishes until 60–70% confluence, incubated with or without 80 µM LPA for 24 h, harvested, centrifuged and re-suspended in fresh medium at 100,000 cell/ml (for MCA electron microscopy) or 5,000 cell/ml (for ex vivo peritoneal adhesion assay) with or without 80 µM LPA. Droplets (20 μl) were seeded on the inner surface of a 150 × 25 mm tissue culture dish lid. Phosphate-buffered saline (PBS; Corning Cellgro) was added to the lower dish and the lid was gently inverted atop the dish. Cells were incubated at 37 °C for 48 h and MCA formation was confirmed by light microscopy.

### Mouse intraperitoneal injections

C57Bl/6 female mice (n = 6, Jackson Laboratories, Bar Harbor, ME) were intraperitoneally injected with 1 ml of 80 µM LPA in PBS, 1 ml of PBS or left non-injected. Injections were performed for 5 consecutive days with the injection site (left and right) switched daily. Mice were then euthanized by CO_2_ inhalation and subsequent cervical dislocation, peritoneal explants were dissected from the murine ventral surface and either processed directly for scanning electron microscopy (SEM) or used for an ex vivo peritoneal adhesion assay. All animal procedures were approved by the University of Notre Dame Institutional Animal Care and Use Committee (protocol 17-07-3998) and were carried out in accordance with the regulations of the same.

### Ex vivo* cell/MCA peritoneal adhesion assay*

Cell/MCA adhesion to murine peritoneum was assessed using ex vivo explants of intact peritoneal tissue^54, 55^. C57Bl/6 female mice (Jackson Laboratories) were dissected using a ventral midline incision; 4 peritoneal tissue pieces were removed and pinned to the bottom of 24-well dishes pre-coated with optically transparent silicone using Sylgard 184 Silicone Elastomer Kit (Fisher, Waltham, MA). For some experiments, prior to peritoneal tissue extraction, mice were subjected to intraperitoneal injections of 80 µM LPA for 5 consecutive days as detailed above. To examine adhesion of individual cells to peritoneal explants, fluorescently labeled EOC cells (200,000 cell/ml), pre-treated or not treated with 80 µM LPA (designated as 2D+ or 2D−), were applied atop murine peritoneal explants and incubated ex vivo for 30 min, 1 and 2 h, as indicated. For peritoneal adhesion of MCAs, fluorescently tagged non-treated (2D − 3D−) or LPA-treated (2D + 3D+) clusters were produced as detailed above and incubated atop murine peritoneal explants (480 MCAs per 1 explant). The assay was stopped after 4 h with 3 × 3 min ice-cold PBS washes and cells/peritoneum were imaged with AMG EVOS fluorescence microscope. All assays were performed in quadruplicate with triplicate samples subjected to quantitative and statistical analysis as described below. The fourth explant was subjected to SEM processing and imaging.

### Scanning electron microscopy (SEM)

MCAs were generated via the hanging drop method, collected and fixed in primary fixative solution (2% Glutaraldehyde, 2% Paraformaldehyde in 0.1 M Cacodylate buffer pH 7.35), washed, processed with 1% Osmium tetroxide in 0.1 Cacodylate buffer and dehydrated as published previously^24^. Primary fixation, washing, secondary processing with 2% Osmium tetroxide and dehydration of mouse peritoneal explants was described in^21, 24^. Critical point drying was performed using Autosamdri-931 (Tousimis Research Corporation), samples were placed on carbon stubs, sputter coated with iridium, and examined under FEI-Magellan 400 field emission SEM. Electron micrograph false colorization was applied using Adobe Photoshop CC 2014 software.

### Quantitative image and statistical analysis

For analysis of ex vivo cell and MCA peritoneal adhesion, murine tissues were imaged (six fields of view per each explant that was incubated with single cells or the total area of MCA-seeded tissue, three biological replicates per each experiment), image analysis was performed with ImageJ (free download). The measured number of attached MCAs for each size group (small, 3–10 cells; medium, 11–50 cells; large, > 50 cells) were normalized by the mean number of attached MCAs of the corresponding control (no LPA-treatment). For quantitative analysis of ultrastructural differences between murine peritoneal explants, the length and thickness of the mesothelial microvilli were measured using standard Fiji open source software^56^ measurement tool applied to SEM micrographs, taken in three distinct areas of both left and right peritoneum compartments per each mouse (Supplemental Fig. 2). Microvilli surface density was quantified as the relative area of microvilli fibers in SEM segmented micrographs assessed using standard Fiji measurement tool. For all assays, statistical significance (defined as p < 0.05) was calculated using a two-sided Mann–Whitney U test. Adobe Photoshop CC2014 software was used in figure preparation.

## Supplementary information

Supplementary information
