# Supplementary material for: Lysophosphatidic acid modulates ovarian cancer multicellular aggregate assembly and metastatic dissemination
Source: Sci Rep. 2020 Jul 2;10:10877. doi: 10.1038/s41598-020-67565-7 (PMC7331713; doi:10.1038/s41598-020-67565-7)
Supplement: Supplementary file 1 — Supplementary information [file 41598_2020_67565_MOESM1_ESM.pdf]

SUPPLEMENTAL DATA FOR:

Lysophosphatidic acid modulates ovarian cancer multicellular aggregate assembly and metastatic dissemination

Yuliya Klymenko [1,2], Brandi Bos [2], Leigh Campbell [2], , Elizabeth Loughran [2], Yueying Liu [2], Oleg Kim [2,4], Jing Yang[2] and M. Sharon Stack [2,3]*

[1] Department of Obstetrics & Gynecology, Indiana University School of Medicine, Indianapolis, IN

[2] Harper Cancer Research Institute, University of Notre Dame, South Bend, IN

[3] Department of Chemistry and Biochemistry, University of Notre Dame, Notre Dame, IN

[4] Department of Cell and Developmental Biology, University of Pennsylvania Perelman School of Medicine, Philadelphia, PA

***** Correspondence: sstack@nd.edu; Tel.: +1-574-631-4100 (M.S.S.), University of Notre Dame, Harper Cancer Research Institute, 1234 N Notre Dame Ave., A 200 Harper Hall, South Bend, IN, 46617, USA

Keywords: ovarian cancer, metastasis, lysophosphatidic acid (LPA), multicellular aggregates (MCAs), peritoneum, mesothelium, adhesion

Klymenko et al., Suppl Figure 1

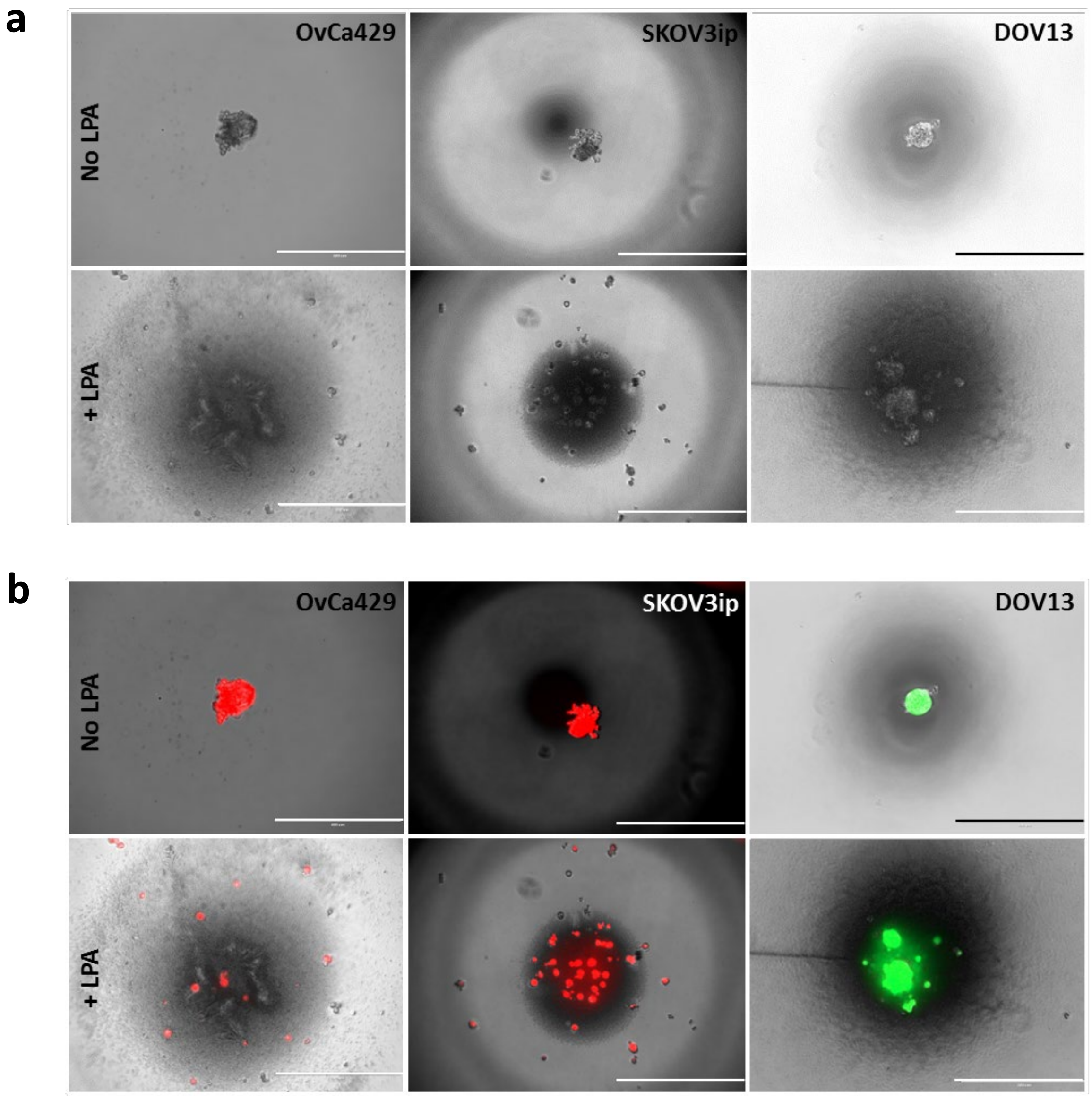

Supplemental Figure 1. Exposure to LPA impairs ovarian cancer MCA assembly. Fluorescently tagged (RFP or GFP) human EOC cells were seeded in 20 µl hanging drops at a 100,000 cell/ml concentration with or without 80µM LPA (Cayman Chemical, Ann Arbor, MI) and incubated for 48-72 hrs. Imaging was performed using AMG EVOS fluorescent microscope and representative MCAs in **A)** transmitted and **B)** merged (transmitted + fluorescent) modes are visualized; scale bar = 400µm.

Klymenko et al, Suppl. Fig. 2

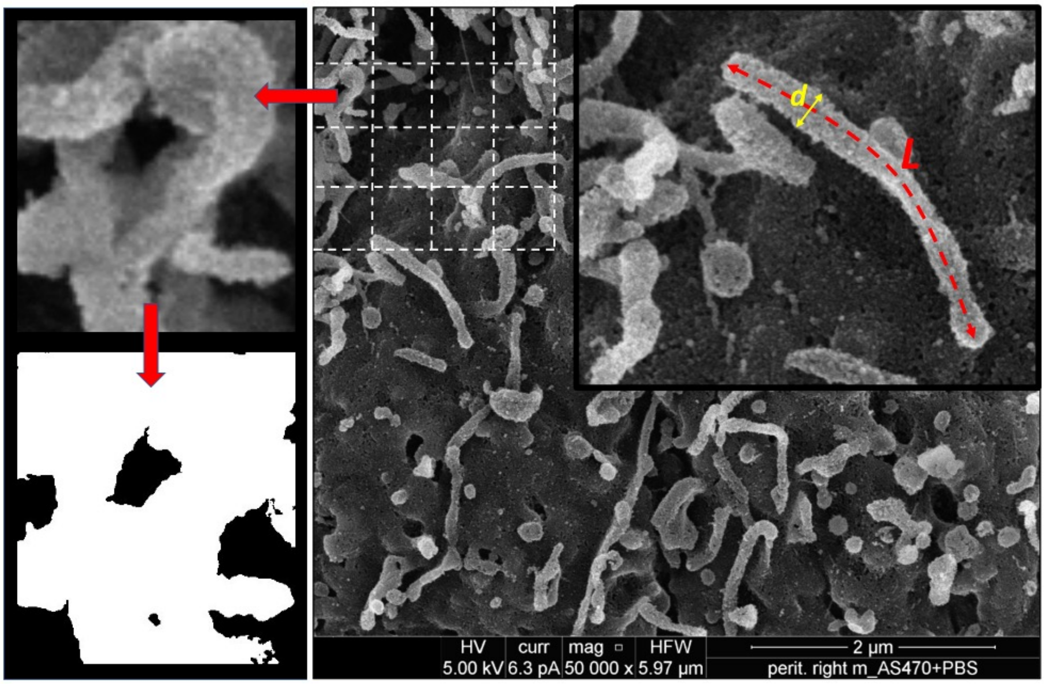

Supplemental Figure 2. Quantitative characterization of mesothelial cells microvilli in *ex vivo* murine peritoneal tissues. A representative SEM image of a peritoneal tissue surface ultrastructure showing mesothelial cells exhibiting multiple microvilli, which were characterized in terms of their length (*L*), thickness (*d*), and relative surface density (s). To calculate the microvilli surface density, each picture was divided into 100 squares (Image J software, National Institutes of Health, Bethesda, Maryland), and the surface density of microvilli of each square was measured from binary images after segmentation of original SEM images using Minimum thresholding (ImageJ). The relative surface density for each square segment was calculated as the ratio of the microvilli area in the square to its total area. The thickness, *d*, (yellow double ended arrow) and length, *L*, (red curved double ended arrow) of the microvilli were evaluated using ImageJ software tools as described in Methods.
